# Understanding the primary health care experiences of individuals who are homeless in non-traditional clinic settings

**DOI:** 10.1186/s12875-022-01932-3

**Published:** 2022-12-27

**Authors:** Jahanett Ramirez, Liana J. Petruzzi, Timothy Mercer, Lauren E. Gulbas, Katherine R. Sebastian, Elizabeth A. Jacobs

**Affiliations:** 1 The Steve Hicks School of Social Work at the University of Texas at Austin, 1925 San Jacinto Blvd, Austin, TX 78712 USA; 2 Department of Population Health, Dell Medical School, The University of Texas at Austin, Austin, TX USA; 3 Department of Internal Medicine, Dell Medical School, The University of Texas at Austin, Austin, TX USA; 4 CommUnityCare Health Centers, Austin, TX USA; 5 Maine Medical Center Research Institute, MaineHealth Institute for Research, Scarborough, ME USA

**Keywords:** People experiencing homelessness, Non-traditional clinic setting, Primary care delivery model, Health care for the homeless, Capability-opportunity-motivation behavior model

## Abstract

**Background:**

Despite the widespread implementation of Health Care for the Homeless programs that focus on comprehensive, integrated delivery systems of health care for people experiencing homelessness, engaging and retaining people experiencing homelessness in primary care remains a challenge. Few studies have looked at the primary care delivery model in non-traditional health care settings to understand the facilitators and barriers to engagement in care. The objective of our study was to explore the clinic encounters of individuals experiencing homelessness receiving care at two different sites served under a single Health Care for the Homeless program.

**Methods:**

Semi-structured interviews were conducted with people experiencing homelessness for an explorative qualitative study. We used convenience sampling to recruit participants who were engaged in primary care at one of two sites: a shelter clinic, *n* = 16, and a mobile clinic located in a church, *n* = 15. We then used an iterative, thematic approach to identify emergent themes and further mapped these onto the Capability-Opportunity-Motivation model.

**Results:**

Care accessibility, quality and integration were themes that were often identified by participants as being important facilitators to care. Psychological capability and capacity became important barriers to care in instances when patients had issues with memory or difficulty with perceiving psychological safety in healthcare settings. Motivation for engaging and continuing in care often came from a team of health care providers using shared decision-making with the patient to facilitate change.

**Conclusion:**

To optimize health care for people experiencing homelessness, clinical interventions should: (1) utilize shared-decision making during the visit, (2) foster a sense of trust, compassion, and acceptance, (3) emphasize continuity of care, including consistent providers and staff, and (4) integrate social services into Health Care for the Homeless sites.

**Supplementary Information:**

The online version contains supplementary material available at 10.1186/s12875-022-01932-3.

## Background

On a single night in 2020, approximately 580,000 people experienced homelessness in the United States [1]. To address the unique needs of both sheltered and unsheltered people experiencing homelessness (PEH), specialized programs addressing healthcare services for the homeless (HCH) have existed since the 1980s [2]. In 2020, 299 health centers received additional federal grant funding through Sect. 330(h) of the Public Health Service Act, to support healthcare for the homeless programs at their sites [3]. Collectively, approximately one million individuals experiencing homelessness are served under this model each year, which focuses on providing integrated care delivery through intensive case management, clinic, and community collaborations [3–6].

The HCH model focuses on providing trauma-informed care by combining both clinical and mental health services to allow for a more integrated and multidisciplinary approach that promotes continuity of care [4]. HCH programs vary in where they are located, how they are structured and how they deliver healthcare and social services [5]. Programs can range from stand-alone homeless healthcare clinics, to shelter-based clinics, mobile clinic vans, drop-in centers, and street medicine teams, with some programs providing multiple care-delivery models of care [5, 6].

However, despite widespread implementation of HCH programs, barriers to care in homeless populations persist and engaging and retaining individuals experiencing homelessness in primary care remains a challenge. Qualitative studies have identified barriers to primary care retention ranging from stigma, distrust of medical professionals, prior negative encounters with rejection, to competing priorities, and logistical complexity [7–9]. Mental illness has also been shown to be a barrier to receiving primary care, with substance abuse, trauma, personality and/or psychiatric disorders contributing to the psychological vulnerability of this population [10–15].

Although several studies have looked at tailored vs. non-tailored primary care experiences of PEH, few have looked at those receiving care in non-traditional settings. To address this gap, we sought to explore the clinic encounters of people experiencing homelessness receiving tailored care at two non-traditional clinic sites served under a single HCH program. In addition, we applied a Capability, Opportunity, Motivation-Behavior (COM-B) model to map their barriers and facilitators to primary care.

The COM-B model identifies three components that influence behavior change: capability, opportunity, and motivation [16]. This model has been used to look at the barriers and facilitators to tobacco use in vulnerable groups, including those experiencing homelessness, in the past [17]. However, to our knowledge, it has not been applied to examine primary care engagement for people experiencing homelessness. By applying the COM-B framework, we were able to structure our way of thinking about facilitators and barriers to care, in a way to better inform the development of future interventions to increase the engagement and retention of people experiencing homelessness in primary care settings.

The primary aim of this study was to explore the facilitators and barriers to engagement and retention in care for individuals experiencing homelessness. By focusing on individuals receiving care in two different, non-traditional, healthcare settings: a mobile clinic in a church location and an integrated clinic inside a homeless shelter, we were able to discern the impact that the setting had on engagement in primary care, when providing homeless-tailored services through a single community health center. In addition, we were able to discern the role shared decision making (SDM) played in promoting continued engagement in care. Shared decision making in this context, was defined as a treatment or management decision that supported the patient’s autonomy, taking into consideration their preferences, goals, and values when presented with options from their health care provider on how to proceed during a clinical encounter [18].

## Methods

### Study design and theoretical framework

This qualitative analysis focused on how the primary care delivery model and HCH setting impacted the healthcare experience of people experiencing homelessness. The primary focus of these exploratory interviews was to better understand how to better address these disparities, by further exploring their engagement and retention in unconventional primary care settings.

We purposely recruited individuals who were engaged in primary care at one of two sites run under one HCH program: a shelter clinic site (SC, *n* = 16) and a church-based clinic site (CC, *n* = 15). We also recruited an additional 11 patients who were not engaged in care, however, these were not included for the purpose of this analysis.

### Population and sample

The data for this study was collected between 2019 and 2020 and the University of Texas at Austin’s Institutional Review Board approved this study. We used convenience sampling to recruit participants at each of the two clinics; they were referred from clinics by health-care staff and community partners, who met the following criteria: English – speaking, aged > 18 years, self-identified as being homeless, and “engaged in care”. Participants were considered “engaged in care” if they had at least two electronic medical record (EMR)-documented visits in the past year, confirmed by healthcare staff. These participants were referred by practitioners and nurses at the clinic who knew them well. Our goal, for this study, was to recruit 15 individuals who were “engaged” in care from each clinic site. We were able to recruit a total of *n* = 31 participants who were engaged in care. They received a $20 gift card for participation. The interviewer was a family-medicine trained physician with experience working with homeless populations in Texas, but was not personally involved in providing care at the clinic sites before, during, or after the participants were recruited.

### HCH site descriptions

Two sites were included in this study: a shelter-based clinic (SC) and a church-based clinic (CC). Both HCH sites provided homeless-tailored services and healthcare staff would occasionally cross-cover both sites. These sites were approximately five miles from each other, although geographically distinct with the shelter-based clinic located in the downtown core, while the church-based clinic was located in the south end of the city. Both provided healthcare through one federally qualified health center (FQHC) network, which provides primary care in underserved areas through Health Resources and Services (HRSA) funding [19]. One site was a clinic housed within a homeless shelter. There was a second homeless shelter next door, as well as several homeless service providers (“soup kitchen”, day resource center, and case management services) at nearby agencies within a few block radius. The shelter also provided some case management and housing navigation services. All of these ancillary services however, were separate from the clinic and were not coordinated together. The shelter-based clinic provided primary care and limited behavioral healthcare (i.e. counseling) to patients on a walk-in basis, as well as through scheduled appointments, five days per week. Appointments were usually 30 min in length, and 8–9 patients were scheduled per half day. The second site was a church-based mobile clinic at a homeless day resource center where patients could receive integrated and coordinated services including housing navigation, supplemental nutrition assistance program (SNAP) or “food stamp” benefits, medical coverage sign-up, mail and ID services, clothing, showers, mental healthcare (psychiatric prescriber and case management) and primary care services. The medical clinic with integrated services was open one day a week and served patients on a walk-in basis only. Appointments lasted approximately 30 min, and between 10 and 15 patients were seen per day by a medical provider, however many more were seen per day by other service providers in the integrated model. Both clinic sites had fully integrated EMR systems as part of a larger FQHC network, and the primary care team consisted of internal medicine trained physicians, nurse practitioners, case managers, and nurses providing services. The medical team had significant experience providing health care to homeless populations. Many had previous experience working with homeless service agencies and team members received on-the-job training, peer mentorship, and continuing education opportunities in homeless-tailored and related services.

### Data collection

We conducted in-depth, semi-structured interviews to understand participants’ primary healthcare experiences, exploring the facilitators and barriers to engagement in primary care. The questionnaire was developed specifically for this study and our questions focused primarily on the nature and characteristics of the care delivery model and clinical setting (Additional File 1). When appropriate, probing was used for clarification and further elaboration of responses. Following the interviews, we collected data on demographics, clinical comorbidities, socioeconomic characteristics, housing status, and payor status. Each interview was audio-recorded, transcribed verbatim, and took approximately 20–30 min to complete. The interview at the church site was conducted in the nave area, separate from others to protect privacy, while the interview at the shelter clinic was conducted in a private office next to patient exam rooms.

### Study analysis

We generated descriptive statistics of the survey results, stratified by group: patients receiving care at the shelter-based clinic and patients receiving care at the church-based mobile clinic. We employed a constant comparative method where we used inductive reasoning to inform our themes [20, 21]. Two coders reviewed ten of the transcripts independently, line-by-line, and met regularly to review, reassess, and refine new codes. Once consensus was reached, these codes were grouped into themes and subthemes, and we generated a coding framework that encompassed all themes across interviews. Thematic analysis was performed until saturation was reached, after no new themes emerged, and coding for all interviews was complete. A COM-B model was then applied, and these themes were mapped onto the six subdomains of the model: physical and psychological capabilities, physical and social opportunities, and automatic and reflexive motivations. In terms of *capability*, this domain encompassed both the physical and psychological factors that contribute to an individual’s ability to bring about a behavior change [16]. *Opportunity* looked at how the environment, time, and other people or organizations could influence behavior change [16]. And *motivation*, looked at both conscious thought processes involving plans or evaluations (reflexive) and habitual processes involving desires and reactions (automatic) [16, 22].

## Results

### Demographics

The median age for those engaged in care at the shelter-based clinic was 59 years old. Most were male (75%), half were African-American (50%), had completed some college or more (50%), and had been homeless for less than 3 years (75%). Most had medical coverage through the county’s local health coverage program called the Medical Access Program (MAP) (69%). In the engaged sample at the church-based clinic, the median age was 49 years old. Most were male (80%), approximately half had less than a high school diploma (40%), most had been homeless for 3 years or less years (60%), and had MAP coverage (80%). See Table 1 for more details.

**Table 1:** Baseline Characteristics of Patients

Characteristics	Engaged at Shelter Clinic (*n* = 16)	Engaged at Church Clinic (*n* = 15)
Median age (range) - years	59 (47–65)	49 (35–62)
Male	75% (12)	80% (12)
Race	44% (7) Non-Hispanic white 50% (8) African American 6% (1) Hispanic / Latino	73% (11) Non-Hispanic white 20% (3) African American 7% (1) Hispanic / Latino
Education	20% (3) Less than High School (HS) 30% (5) Completed HS/GED 50% (8) Some College or more	40% (6) Less than High School (HS) 20% (3) Completed HS/GED 33% (5) Some College or more 7% (1) Did not respond
Homelessness	25% (*n* = 4, less than 1 year) 50% (*n* = 8,1–3 years) 25% (*n* = 4, 4 + years or more)	27% (*n* = 4, less than 1 year) 33% (*n* = 5, 1–3 years) 40% (*n* = 6, 4 + years or more)
Insurance	69% (*n* = 11, MAP insurance) 31% (*n* = 5, Medicare/Medicaid)	80% (*n* = 12, MAP insurance) 13% (*n* = 2, Medicare/Medicaid) 7% (*n* = 1, No response)

#### Capability: physical and psychological

Among other challenges, people experiencing homelessness face stark health disparities due to structural barriers to housing, healthcare, and income [13, 14]. In the primary care context, participants mentioned physical and psychological factors that led to engagement in care or barriers to obtaining care. In one example, at the church-based clinic, a participant was able to obtain care when one of the nurses facilitated medical attention:

*I came here one Thursday, not even thinking about going to the clinic and I had the right, not the right, the left side of my nose, all bruised and inflamed and in pain. And the nurse that knows me really well, she came outside and I asked her, “Does this look bad to you?“ And she’s like, “Yeah, I’m going to put you right away on the clinic list.“ And she was able to get me in before anybody else. .. I found out I had a slight infection in my nose. (CC)*

In this instance, the participant was able to engage in care due to several factors: the familiarity of the participant with the clinic and staff, the recognition of a medical issue that needed immediate attention by the nurse, and the capability of the clinic to accommodate emergencies. This is an example of how the participant’s ability to seek care [physical capability], combined with the clinic’s ability to provide that care [physical opportunity] facilitated the patient’s engagement in primary rather than emergency care for an infection that needed prompt medical attention.

Psychological capability and capacity became important barriers to care in instances when patients had issues with memory or difficulty with perceiving psychological safety in healthcare settings. The following example demonstrates how memory difficulties could interfere with continuity of care:

*I mean, the appointments are set a little further behind than I would like. And sometimes it’s an inconvenience meeting these appointments because they set so far behind I actually forget about them. (SC)*

Cognitive impairment played an important role in the decision making of individuals. In addition, to forgetting appointments, if they were spaced too far apart, people experiencing homelessness sometimes had to make significant changes to their routines to accommodate for memory difficulties:

*Because I’ve got early-onset dementia as one of my permanent side effects from the surgeries...That’s why I stay around here a lot and I walk around here a lot because it’s repetition, it’s familiarity versus if I get on the bus I feel like I’m...I’m just going to freak out and I’m going to have a dementia episode...I need familiarity and repetition and [the church based clinic]is that familiarity and repetition for me because I’m always coming here every day. (CC)*

In terms of psychological safety, in the context of healthcare, asking questions, speaking up and making suggestions, without the fear of retribution, is seen as essential in promoting patient safety and team performance [23]. At the individual level, positive indicators of psychological safety include having a voice, learning, support, and familiarity [24]. Interpersonal factors such as trust, respect, effective relationships, and high-quality communication also play a role in creating an environment of psychological safety [24, 25]. These same indicators were identified by the participants as fostering a conducive environment for engagement in primary care for populations experiencing homelessness. As the example below shows, psychological safety was essential for successful team-based care in HCH settings:

*I know a lot of my friends that come here, I don’t like to say ‘homeless people’ we’re all friends and family here. They feel the same way. They don’t like seeing a different doctor every week because it’s just not... It doesn’t make them feel secure and being able to tell somebody else their health issues. (CC)*

#### Opportunity : physical and social

Care integration, or the physical presence of multiple agencies, providers, and services in one clinic at one time, was particularly useful for engaging PEH. As the example below shows, some participants first engaged in social services, and then received primary care treatment after learning about the clinic. Once engaged, they valued having access to multiple health and social service agencies under one roof:

*This is the first place I found it has this much going underneath the roof. You can get something to eat, a good cup of coffee. I actually had just come down here to see about the day labor. (CC)*

This is particularly important for PEH as they have multiple health and social needs, so coordination of services is crucial to ensure that health professionals are not duplicating efforts:

*I think it’s a really good setup. I honestly love the fact that all the agencies are here. It’s not just one. .. I feel like it’s a very beautiful collaboration where people from different agencies truly know each other. (CC)*

In terms of social opportunity, it is also important to have awareness of how the setting of the clinic could potentially act as a facilitator to undesirable behaviors. For example, at the shelter site, providing alternative ways to reach providers was especially important, due to illicit drug use happening around the shelter. To enter the clinic inside the shelter, patients first had to form long lines through security, with other people who were not accessing the clinic. As one participant stated:

*The location is fine, but the activities is cutting down, so it will be perfect when the authorities get this chuck cleaned up outside.. . Well, they sell drugs, and they drink. You well I can’t really say nothing, I do some of it myself you know. But I drink, smoke weed, but if that weren’t out there I probably wouldn’t do it as much. (SC)*

#### Motivation: reflexive and automatic

In the context of primary care, motivation for engaging and continuing in care often came from a team of providers and nurses working together with the patient to facilitate change. Below is an example of how the provider’s encouragement led to positive behavior change, despite setbacks.

*I just came through a three-month thing with Hep-C, and I just took the last tablet a couple days ago. Throughout the course of that, I had stopped taking it, because I thought it was giving me a side effect. The doctor came in and encouraged me to keep continuing it, so I’ve continued it, and I’ve started feeling better. They ran a small test to see that it’s not detected anymore…If it wasn’t for them encouraging me, and if they didn’t care, I would’ve stopped it. If they said, “Okay, that’s your choice”. But they just kept me going! So, they’re my team, and I appreciate them. (SC)*

Although the patient had stopped his medication due to side effects, the provider’s reassurance allowed him to re-evaluate his thought process involving his plan to continue the medication (reflexive motivation), thus enabling him to complete the treatment successfully. Education also played an important role in motivation:

*The medication, the kindness about and understanding about how I don’t like to take the meds, but you know, they’ve truly given me explanations that make me want to take them. Because it’s really dire need for me to have those meds. (SC)*

It is important, however, to keep the patient’s desires and affective responses in mind (automatic motivation), as acting proactively, instead of collaboratively, could be interpreted negatively by the patient:

*Well one thing, they don’t need to set you an appointment to go do something unless you want to. Don’t say, “Well, I’ve already got your appointment set up. You just go.“ You didn’t even ask me if I wanted to go do it in the first place. Let the patient agree to doing that and don’t just say, “I got it done for you,“ and “It’s all set up for you.“ You didn’t even ask me for my opinion. (CC)*

Shared-decision making (SDM) can help empower vulnerable populations receiving care by directly addressing communication challenges and patient preferences that can negatively affect the clinical encounter. In this case, shared-decision making with the patient could have be used to avoid misunderstandings.

## Discussion

This study used the three domains of the COM-B model, capability, opportunity, and motivation, to understand the engagement of people experiencing homelessness in primary care. Care accessibility, quality and integration were capability themes that were often identified by participants. While all of these factors may be relevant to healthcare engagement and retention more broadly, they are particularly important for PEH. Our findings suggest that although the HCH model has several characteristics that contribute to patient satisfaction and healthcare participation among PEH, there are areas of improvement that can be addressed to enhance care.

Patients who were engaged in care, specifically in a church-based clinic, valued both the healthcare personnel, as well as the staff from the navigation center and the other social service agencies. Participants mentioned the importance of having the center staff proactively working with the healthcare staff to identify patients needing medical care, and playing an important role in supporting their continuity of care. The relationship between patients and their providers was also highly regarded and provided the motivation for continued engagement in care. Patients preferred to have the same provider every time, both for convenience (not having to tell their story repeatedly), and as a matter of trust (they presumed their provider to have their best interests in mind). In addition, feelings of security, inclusiveness, and integration of services were important in both drawing patients to the clinic, as well as facilitating and maintaining their engagement with primary care. This is consistent with prior research, which has shown trust and safety in healthcare settings to be highly valued among people experiencing homelessness, often leading to an increased likelihood of seeking care and adhering to treatment recommendations [26–28]. In particular, consistency in terms of the providers that were available was important, as trust was difficult to build and maintain among the homeless population.

Patients were also able to identify areas of improvement in settings offering HCH services. A medical clinic in a shelter was seen as an asset when it came to eliminating transportation barriers, as patients could easily walk from the shelter to the clinic within the building. However, as an example of negative “social opportunity”, illicit drug use around the shelter was a significant concern, as it could trigger a relapse, and made it difficult for some patients to remain sober. In addition, patients commented on the need for separate entrances for the clinic and the shelter, to make it easier to access services when they were coming from outside. To enter the shelter, patients often formed long lines outside the building and had to go through security and a metal detector prior to being allowed inside. This sometimes led to negative encounters with other people accessing the shelter, for reasons other than healthcare services.

Other suggestions to improving services included having the clinic appointments scheduled more regularly. Patients cited difficulty with remembering appointments, particularly if they were scheduled too far in advance. They also valued being able to exercise their autonomic motivation by participating in shared-decision making over having providers making decisions for them, both in terms of medications, and scheduling further work-up. They appreciated having their medications available to them at the time of their appointment for easy access and having the nursing staff review their instructions after seeing the physician.

Lastly, having several services available under one roof was also an important consideration. In addition to the barriers already mentioned, it also reduced the need to coordinate transportation multiple times, as this could be particularly difficult. Participants stated that having access to a bus pass was essential for accessing healthcare and social services and they valued having medical professionals who are aware of homeless-specific needs.

Of note, because the participants who were engaged in care were recruited at the time when they were seeking services, it was necessary to conduct the interviews at the location where they received care. This could potentially have led to participants feeling hesitant to share negative feedback about their clinic experience or provider, and subsequently emphasize positive encounters. However, all participants were given an opportunity to reflect on how their overall experience in the clinic could be improved and were reminded that their feedback, both positive and negative, was important to advancing healthcare for people experiencing homelessness. In addition, the interviews were conducted in private areas within the clinic out of earshot to other clinical staff, to protect patient confidentiality, and minimize bias.

### Limitations/future directions

There are several limitations to consider when interpreting the findings from this study. First, the sample was predominantly male (77%), although this is somewhat representative of the homeless population in general [3, 28]. Similarly, there was a limited number of Hispanic participants (6%), which was significantly lower than the Austin homeless population (26.5%) [29]. Second, this study utilized convenience sampling of individuals at two different clinic sites, and may not be representative of the larger Austin homeless population. However, this is a common limitation for qualitative studies and does not diminish the importance of the study findings. Third, this analysis only included PEH that are engaged in primary care, which may have influenced our findings. Lastly, this study took place within a county that offers a relatively comprehensive indigent healthcare plan, which may have influenced more favorable access to care. Not all communities have access to this level of healthcare coverage for those who have limited income or are experiencing homelessness. However, it is important to note that Medicaid expansion has not been passed in Texas, and this could have a significant impact on barriers to care for homeless individuals in Austin and across the state.

Future studies should explore the unique perspectives of people of color experiencing homelessness, particularly Black and Hispanic populations. Black adults represent approximately 40% of the homeless population, but only 12% of the overall population [3]. Black adults are similarly disproportionately represented in Austin’s homeless population [29]. Hispanic adults represent 23% of the homeless population but only 16% of the overall population [3]. Further, Hispanic adults have almost twice the lifetime rate of homelessness compared to non-Hispanic Whites (8.1% vs. 4.8%) [30]. Therefore, more research is needed to understand the unique needs of Black and Hispanic adults experiencing homelessness. Additionally, it could be beneficial to explore how Medicaid expansion has impacted barriers and facilitators to healthcare for homeless individuals through qualitative analysis. Several quantitative analyses have suggested an increase in office visits as well as emergency room visits among homeless individuals due to Medicaid expansion, but more data is needed [31, 32]. Lastly, it will be important for future studies to continue to explore how COVID-19 has impacted access to healthcare for homeless individuals.

## Conclusion

Patients experiencing homelessness have unique needs and psychosocial stressors that require sensitivity from primary care providers and staff. Convenience of services are essential, such as location, short clinic wait times and co-location of services. Clinical interventions should: (1) utilize shared-decision making during the visit, (2) foster a sense of trust, compassion, and acceptance, (3) emphasize continuity of care, including consistent providers and staff, and (4) integrate social services into HCH sites, so that individuals experiencing homelessness can receive wrap-around services to address their multifaceted needs such as housing, employment, food insecurity and/or mental health support.

## Supplementary Information

**Additional file 1. **Qualitative Interview Questions.

## Data Availability

The datasets used and/or analyzed during the current study are de-identified and available from the corresponding author on reasonable request.

## Abbreviations

PEH: People Experiencing Homelessness
HCH: Health Care for the Homeless
COM-B: Capability, Opportunity, Motivation, Behavior
SDM: Shared decision-making
SC: Shelter Clinic
CC: Church Clinic

## References

[1] Health Resources & Services Administration. 2020 Special Populations Funded Programs. Available at: https://data.hrsa.gov/tools/data-reporting/special-populations. Accessed on 25 Aug 2021.

[2] Zlotnick C, Zerger S, Wolfe PA (2013). Health Care for the Homeless: what we learned in the past 30 years and what’s Next. Am J Public Health.

[3] U.S. Department of Housing and Human Development. The 2020 Annual Homeless Assessment Report (AHAR) to congress. Available at: https://www.huduser.gov/portal/sites/default/files/pdf/2020-AHAR-Part-1.pdf. Accessed on 25 Aug 2021.

[4] National Health Care for the Homeless Council. Demonstrating value: measuring the value and impact of the health care for the homeless grantees. Available at: https://nhchc.org/wp-content/uploads/2019/08/hch-value-paper-1.pdf. Accessed on 25 Aug 2021.

[5] National Association of Community Health Centers. Health Care for the Homeless. Available at: https://www.nachc.org/health-center-issues/special-populations/health-care-for-the-homeless/#:~:text=Program%20Background&text=The%20Patient%20Protection%20and%20Affordable,expand%20services%20and%20deliver%20sites.&text=In%202017%2C%20299%20health%20centers,nearly%20one%20million%20homeless%20patients. Accessed on 25 Aug 2021.

[6] National Health Care for the Homeless Council. The Health Care for the Homeless Program. Available at: https://nhchc.org/wp-content/uploads/2021/04/HCH-Fact-Sheet_2021.pdf. Accessed on 25 Aug 2021.

[7] Varley AL, Montgomery AE, Steward J (2020). Exploring quality of primary care for patients who experience homelessness and the Clinicians who serve them: what are their aspirations?. Qual Health Res.

[8] McCallum R, Medved MI, Hiebert-Murphy D, Distasio J, Sareen J, Chateau D (2020). Fixed nodes of transience: narratives of homelessness and Emergency Department Use. Qual Health Res.

[9] Moore-Nadler M, Clanton C, Roussel L (2020). Storytelling to capture the Health Care Perspective of People who are homeless. Qual Health Res.

[10] White BM, Logan A, Magwood GS (2016). Access to Diabetes Care for populations experiencing homelessness: an Integrated Review. Curr Diab Rep.

[11] Koh KA (2020). Psychiatry on the Streets – caring for homeless patients. JAMA Psychiatry.

[12] Fazel S, Khosla V, Doll H, Geddes J (2008). The prevalence of Mental Disorders among the Homeless in Western Countries: systematic review and Meta-regression analysis. PLoS Med.

[13] Upshur CC, Jenkins D, Weinreb L, Gelberg L, Orvek EA (2018). Homeless women’s service use, barriers, and motivation for participating in substance use treatment. Am J Drug Alcohol Abuse.

[14] Chrystal JG, Glover DL, Young AS (2015). Experience of primary care among homeless individuals with mental health conditions. PLoS One.

[15] Patanwala M, Tieu L, Ponath C, Guzman D, Ritchie CS, Kushel M, Physical (2017). Psychological, Social, and existential symptoms in older homeless-experienced adults: an observational study of the Hope Home Cohort. J Gen Intern Med.

[16] Michie S, van Stralen MM, West R (2011). The behavior change wheel: a new method for characterizing and designing behavior change interventions. Implement Sci.

[17] Gentry S, Forouhi NG, Notely C (2019). Are electronic cigarettes an effective aid to smoking cessation or reduction among vulnerable groups? A systematic review of quantitative and qualitative evidence. Nicotine Tob Res.

[18] Tonelli M, Sullivan M (2019). Person-centered Shared decision making. J Eval Clin Pract.

[19] Health Resources & Services Administration. Federally qualified health centers. Available at: https://www.hrsa.gov/opa/eligibility-and-registration/health-centers/fqhc/index.html. Accessed 6 Jun 2022.

[20] Olson JD, McAllister C, Grinnell L, Walters KG (2016). Applying constant comparative method with multiple investigators and Inter-Coder Reliability. Qualitative Rep.

[21] Boeije H (2002). A Purposeful Approach to the constant comparative method in the analysis of qualitative interviews. Qual Quantity.

[22] Nickbakht M, Meyer CJ, Saulsman L (2022). Barriers and facilitators to asking adults with hearing loss about their emotional and psychological well-being: a COM-B analysis. Int J Audiol.

[23] O’Donovan R, Van Dun D, McAuliffe E (2020). Measuring psychological safety in healthcare teams: developing an observational measure to complement survey methods. BMC Med Res Methodol.

[24] O’Donovan R, McAuliffe E (2020). Exploring psychological safety in healthcare teams to inform the development of interventions: combining observational, survey and interview data. BMC Health Serv Res.

[25] Ito A, Sato K, Yumoto Y, Sasaki M, Ogata Y (2022). A concept analysis of psychological safety: further understanding for application to health care. Nurs Open.

[26] Feldman BJ, Kim JS, Mosqueda L (2021). From the hospital to the streets: bringing care to the unsheltered homeless in Los Angeles. Health care.

[27] Magwood O, Leki VY, Kpade V (2019). Common trust and personal safety issues: a systematic review on the acceptability of health and social interventions for persons with lived experience of homelessness. PLoS One.

[28] Purkey E, MacKenzie M (2019). Experience of healthcare among the homeless and vulnerably housed a qualitative study: opportunities for equity-oriented care. Inter J Equity Health.

[29] Ending Community Homelessness Coalition. 2021 Racial Disparities Report. Available at: https://www.austinecho.org/wp-content/uploads/2021/09/2021-Racial-Disparities-Report_FINAL.pdf. Accessed on 19 Dec 2022.

[30] Fusaro VA, Levy HG, Shaefer HL (2018). Racial and ethnic disparities in the lifetime prevalence of homelessness in the United States. Demography.

[31] Kaiser Family Foundation. Early impacts of the medicaid expansion for the homeless population. Available at: https://www.kff.org/uninsured/issue-brief/early-impacts-of-the-medicaid-expansion-for-the-homeless-population. Accessed on 27 Aug 2021.

[32] Self J, Callison K, Goudie A, Lewis K, Thompson J (2021). Medicaid Expansion and Health Services use for adults experiencing homelessness in Arkansas: study examines Medicaid expansion and health care use among homeless adults in Arkansas. Health Aff.

