# Supplementary material for: Understanding the primary health care experiences of individuals who are homeless in non-traditional clinic settings
Source: BMC Prim Care. 2022 Dec 27;23:338. doi: 10.1186/s12875-022-01932-3 (PMC9792313; doi:10.1186/s12875-022-01932-3)
Supplement: Supplementary file 1 — Additional file 1. Qualitative Interview Questions. [file 12875_2022_1932_MOESM1_ESM.docx]

**Additional File 1: Qualitative Interview Questions**

1. What does healthcare mean to you?

2. What has been your experience like been when you need to see a doctor?

3. What keeps you and/or people you know from going to get care if you need it?

4. What is the most important factor you consider when deciding where to go for healthcare?

5. What is it you like/don’t like about getting health care in this current setting?

6. Is there anything about the providers/team you like/don’t like?

7. How could the location or setting where care is delivered be improved?

8. How could the care you receive be improved?

9. What keeps you coming back to this clinic?

10. Do you have hypertension or cardiovascular disease?

11. If the clinic were to start offering group visits for heart care, would you be interested in

participating?

12. What do you thing is the most important thing or things for doctors to keep in mind when they

someone experiencing homelessness?

13. Anything else you would like to share with us?
